# T3 and T4 autoantibodies: emerging biomarkers for evaluating thyroid disorders

**DOI:** 10.3389/fendo.2025.1537222

**Published:** 2025-06-12

**Authors:** Jiameng Liu, Chaoming Mao, Xueqian Mao, Xi Wang, Tingting Zheng, Liyang Dong, Yufei Mao

**Affiliations:** ^1^ Department of Nuclear Medicine, The Affiliated Hospital of Jiangsu University, Zhenjiang, Jiangsu, China; ^2^ Department of Ultrasound Medicine, The Affiliated Hospital of Jiangsu University, Zhenjiang, Jiangsu, China

**Keywords:** thyroid hormone autoantibodies, immune checkpoint blockade immunotherapy, development of assay kits, biomarker, immunoassay

## Abstract

**Introduction and objectives:**

The clinical significance of thyroid hormone autoantibodies, specifically triiodothyronine autoantibodies (T3-Ab) and thyroxine autoantibodies (T4-Ab), is not well understood due to current detection method limitations. This study investigated the clinical utility of T3-Ab and T4-Ab as biomarkers for thyroid function by developing a Magnetic Chemiluminescent Immunoassay (MCLIA) kit.

**Methods:**

A chemiluminescent immunoassay kit was developed using magnetic nanomicroparticles conjugated with T3 or T4 antigens. An indirect detection approach (magnetic microparticle antigen-target antibody-anti-human IgG antibody) was employed. Reference ranges were established using 415 serum samples from healthy individuals. Additionally, serum samples from 1,654 patients with various diseases were analyzed for T3-Ab and T4-Ab distribution levels and positive rates. Mass spectrometry and recovery experiments assessed potential interference of T3-Ab and T4-Ab with thyroid hormone detection.

**Results:**

The validation process confirmed the efficacy of the MCLIA kit in detecting serum T3-Ab and T4-Ab. The reference ranges for both antibodies were set at ≤ 1.0 AU/mL and showed no significant correlations with other thyroid markers, including FT3, FT4, TSH, TG, TG-Ab, TPO-Ab, or TR-Ab. Notably, T3-Ab and T4-Ab levels interfered with FT3 and FT4 detection, especially in competitive chemiluminescent immunoassays. Elevated levels of T3-Ab and T4-Ab were found in patients undergoing immune checkpoint blockade therapy.

**Conclusions:**

This study presents the first MCLIA kit for detecting T3-Ab and T4-Ab in human serum, revealing their potential as thyroid disorder biomarkers, particularly in cancer patients undergoing immune checkpoint blockade therapy, where they interfere with thyroid hormone measurements.

## Introduction

The thyroid gland is a crucial organ within the endocrine system that plays a vital role in the regulation of various physiological functions. Imbalances in thyroid function can significantly disrupt endocrine operations, as thyroid hormones are essential for cardiovascular health and metabolic processes (1, 2). Recent studies indicate a growing prevalence of thyroid dysfunction, which is closely associated with modern lifestyle and dietary habits, raising concerns about its negative impact on patients’ overall health and quality of life. In clinical laboratories, the assessment of thyroid function relies primarily on immunoassay techniques to measure the serum levels of thyroid-stimulating hormone (TSH) and thyroid hormones (3, 4). While these immunoassays are favored due to their automation, rapid turnaround times, and high specificity, they can produce nonspecific interferences leading to false-positive or false-negative results (5). Therefore, the ability to quickly and accurately detect such interferences is crucial.

Discrepancies often exist between thyroid function test results and clinical symptoms, along with irregular levels of TSH, free triiodothyronine (FT3), and free thyroxine (FT4). As a result, laboratory personnel must carefully identify and exclude potential interfering factors to ensure accurate diagnoses. Notable sources of interference in T3/FT3 and T4/FT4 measurements include endogenous factors that nonspecifically bind to assay reagents, such as heterophile antibodies; albumin mutations linked to familial dysalbuminemic hyperthyroxinemia; thyroid hormone autoantibodies (THAb); and macro-TSH (6–9). The literature has focused primarily on the detection of these interferences via radioimmunoassays, which often underplay the detection of thyroid hormone autoantibodies compared with other thyroid antibodies. THAbs, comprising subclasses such as anti-T3 IgG, anti-T3 IgM, anti-T4 IgG, and anti-T4 IgM, predominantly feature the IgG type (10).

Traditionally considered nonimmunogenic, thyroid hormones are viewed as haptens, with thyroglobulin potentially triggering autoimmune responses. However, the mechanisms behind the production of endogenous thyroid hormone antibodies remain unclear. Epidemiological data indicate that the prevalence of thyroid hormone autoantibodies in healthy individuals varies from 0% to 25%, highlighting the need for standardized detection methodologies (11, 12). The clinical relevance of these autoantibodies in various diseases has yet to be thoroughly investigated, necessitating the development of robust assay kits. Currently, despite advancements in nonradioactive labeling techniques such as chemiluminescent immunoassays, the absence of commercial kits for T3-Ab and T4-Ab detection restricts their clinical applicability. This study aims to address this gap by developing magnetic chemiluminescent immunoassay (MCLIA) kits for the serum detection of T3-Ab and T4-Ab, with the goal of providing preliminary insights into their clinical utility.

## Materials and methods

### T3-Ab and T4-Ab MCLIA protocol

**Figure 1:**
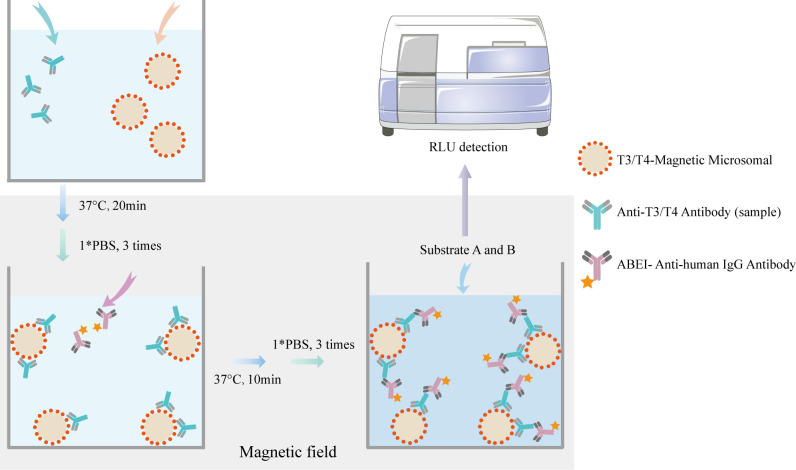
Principle of MCLIA for detecting T3-Ab and T4-Ab. The T3/T4 antigen-coated chemibeads, T3-Ab/T4-Ab, and ABEl Anti-human lgG antibody formed a complex.

An indirect MCLIA was performed to measure the serum levels of T3-Ab and T4-Ab, as illustrated in **Figure 1**. The entire testing process took 35 min, with the following protocol: 10 μL of serum sample, calibration standard, or quality control sample was added to each reaction cup. Next, 20 μL of T3/T4 antigen-coated magnetic beads (with a ratio of 1 mg of beads to 2 μg of antigen) and 150 μL of reaction buffer (100 mmol/L PBS) were introduced into the reaction cup. The mixture was incubated at 37°C for 20 min. After three washes with PBS, 200 μL of ABEI-labeled anti-human IgG (with a ratio of 1 mg of ABEI to 200 μg of anti-human IgG) was andded and the mixture was incubated for an additional 10 min at 37°C. Following three more PBS washes, a chemiluminescent substrate (Snibe, China) was added, consisting of 200 μL of solution A (sodium bicarbonate solution) and 200 μL of solution B (peroxide solution) was added. The luminescent signal was measured via a MAGLUMI X8 (Snibe, China) after a 5 min incubation.

### Apparatus and chemicals

The T3 and T4 antigens, magnetic particles, T3-Ab and T4-Ab calibrators, PBS, aminobenzenesulfonyl isocyanate (ABEI), anti-human IgG, substrates A and B, reaction cups, and the high-throughput chemiluminescence immunoanalyzer (MAGLUMI X8) utilized in the MCLIA tests were all obtained from Snibe.

### Patients and serum collection

The sera of 2069 individuals from Jiangsu University were evaluated, comprising 415 healthy individuals and 1654 patients with various conditions. The patient cohort (n = 1654) consisted of individuals aged 25-85 years (mean age 50 ± 8 years), with 47% male and 53% female participants. The healthy control group included age-matched (20-75 years, 45 ± 10 years) volunteers with balanced gender distribution (42% male, 58% female) and no history of psychiatric/neurological disorders. This patient cohort consisted of 115 patients with type 2 diabetes, 27 with renal insufficiency, 31 with chronic gastritis, 154 pregnant patients, 177 with coronary atherosclerosis, 62 with hypertension, 37 with acute myocardial infarction, 46 with cardiac insufficiency, 58 with atrial fibrillation, 70 with arrhythmia, 33 with unstable angina, 103 with Hashimoto’s thyroiditis, 53 with Graves’ disease, 105 with thyroid nodules, 51 with hyperthyrea, 70 with hypothyrea, 255 with tumors (nonimmunotherapy), and 207 with tumors (immune checkpoint blockade therapy). The remaining serum samples from routine laboratory tests were collected, and the separated sera were promptly stored at -80°C for later analysis. The control group underwent consistent sample collection and processing procedures as the patient group, and the same control group was used for comparative analyses with the patient group throughout the entire study.

The sample sizes for subgroup analyses were based on real-world data available from clinical laboratories. For exploratory analyses (such as gender and age stratification), sample sizes were determined by the actual enrolled healthy control population (n = 415), aiming to provide descriptive evidence. For predefined inter-group comparisons, sample sizes were determined through power analysis: assuming an effect size of Cohen’s d = 0.8 for T3-Ab/T4-Ab concentration differences, calculations indicated that n = 35 cases per group were required to achieve 80% power (α = 0.05, two-tailed test). Ultimately, the number of cases in the majority of groups included exceeded 35, with the actual power exceeding 80%, ensuring the reliability of confirmatory analyses.

### Construction of the MCLIA calibration curve

To construct the T3-Ab and T4-Ab calibration curves, T3-Ab and T4-Ab IgG solutions were serially diluted to concentrations of 0, 0.2, 0.4, 0.8, 1.6, 3.2, 6.4, 12.8, 25.6, and 50 AU/mL. The relative luminescence units (RLU) for each standard concentration were measured via the MAGLUMI X8 (Snibe, China), following standard detection protocols. The data points were plotted in a scatter plot of the RLU values against their corresponding concentrations. Linear regression analysis was performed via GraphPad Prism v8.0 software to determine the regression equations and coefficients.

### Statistical analysis

Statistical analyses and graphical representations were performed via GraphPad Prism v8.0 (GraphPad Software Inc.). The nonparametric Mann–Whitney U test was applied for comparisons between two groups, whereas one-way analysis of variance (ANOVA) followed by the Tukey–Kramer multiple comparison test was used for comparisons among multiple groups. Correlation analyses were conducted via the Pearson and Spearman correlation methods. A significance level of *p* < 0.05 was set to determine statistical significance.

## Results

### Calibration curves

**Figure 2:**
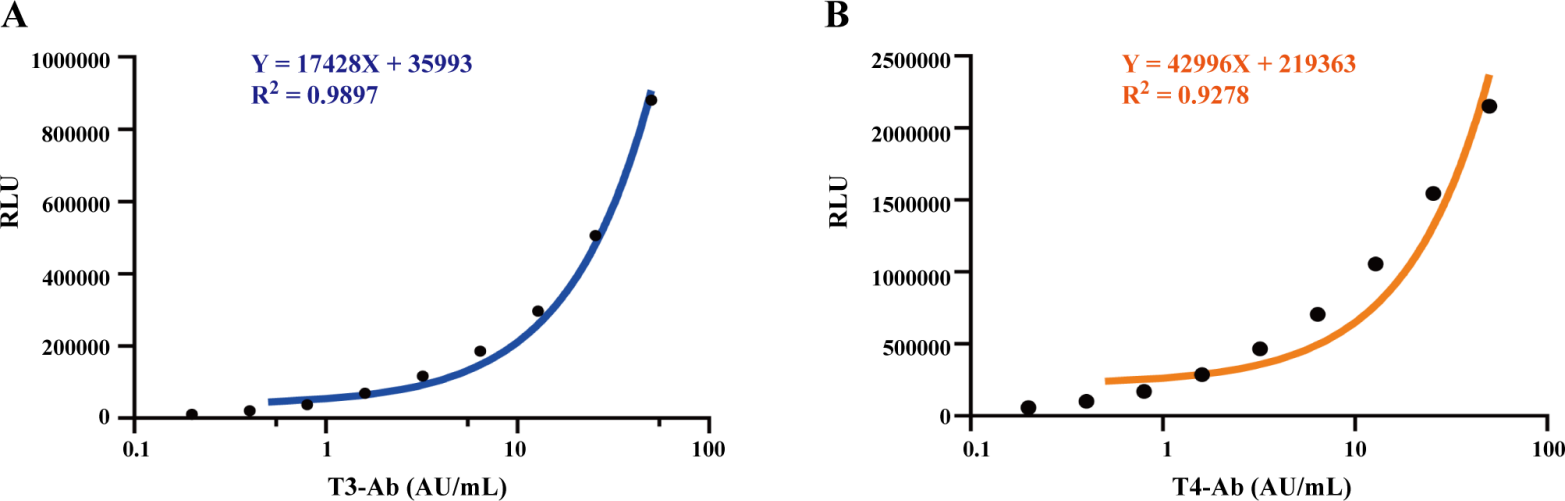
Establish a calibration curve. **(A, B)** Calibration curves of MCLIA for detecting T3-Ab and T4-Ab in human serum. Each point represents the mean of replicate measurements (n = 3).

The calibration curves for T3-Ab and T4-Ab are shown in **Figure 2**. The MCLIA signal exhibited a linear increase in response to increasing concentrations of T3-Ab and T4-Ab, enabling the generation of the calibration curves (**Figure 2A**: Y = 17428X + 35993, R² = 0.9897; **Figure 2B**: Y = 42996X + 219363, R² = 0.9278). The standard curves revealed concentration ranges of T3-Ab and T4-Ab from 0 to 50 AU/mL, corresponding to chemiluminescence signals between 35993–907393 and 219363–2369163, respectively.

### Establishment of the reference value range

**Figure 3:**
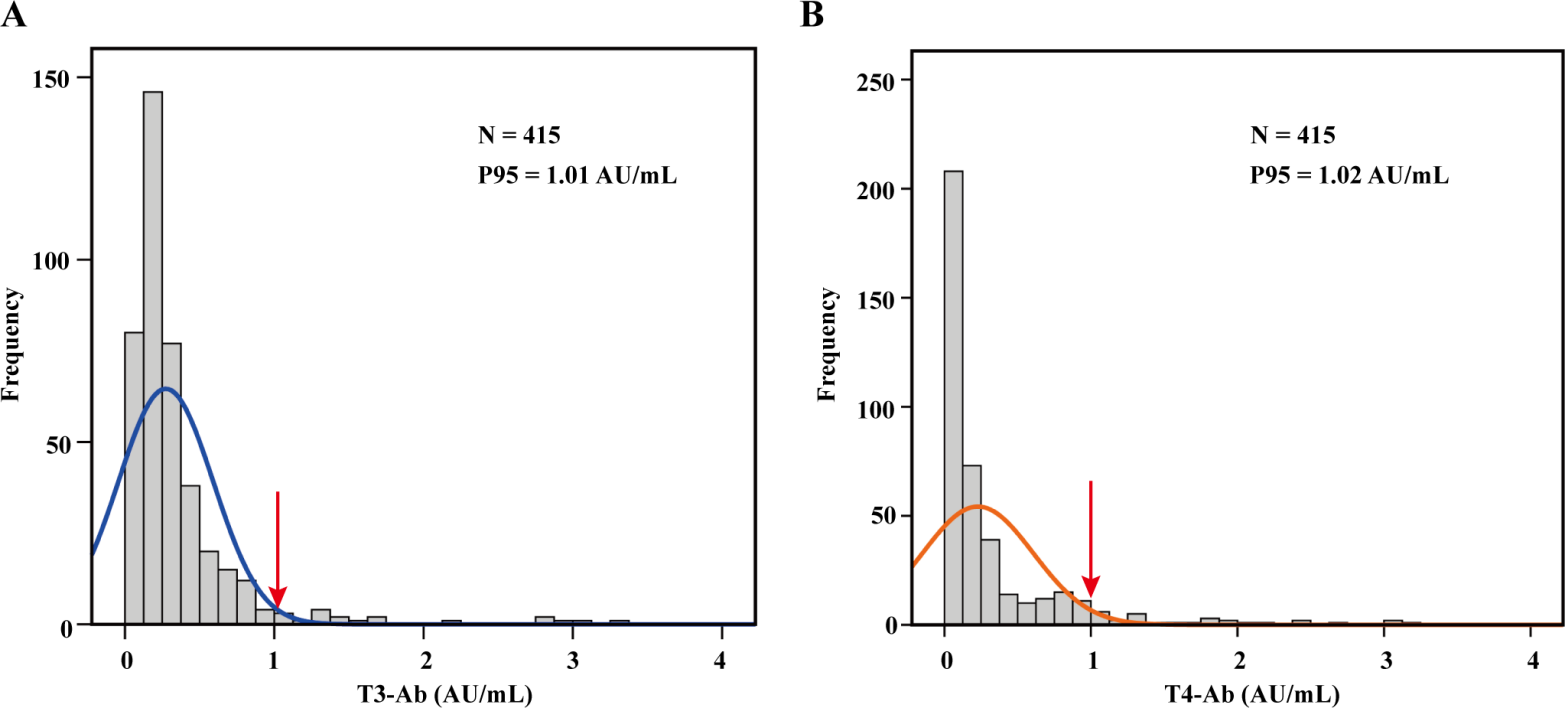
Establishment of the reference range. **(A, B)** Histograms illustrating the distribution of T3-Ab and T4-Ab across 415 healthy individuals, with fitted curves. A one-sided reference range was established based on the P95 distribution. Data analysis was performed via SPSS 17.0.

To establish reference ranges for T3-Ab and T4-Ab, we examined serum samples from 415 healthy individuals (173 males and 242 females). The data were analyzed for frequency distribution, resulting in the creation of histograms and fitting curves. The Kolmogorov-Smirnov test confirmed that T3-Ab and T4-Ab levels followed a nonnormal distribution in the healthy population. As shown in **Figure 3**, we utilized percentiles for data ranking; since low antibody values lack clinical relevance, we adopted the one-sided 95th percentile as the medical reference range. Specifically, T3-Ab concentration was determined to be 1.01 AU/mL, and T4-Ab concentration was accordingly set at 1.02 AU/mL. For the purpose of further statistical analyses, a T3-Ab ≤ 1.0 AU/mL and a T4-Ab ≤ 1.0 AU/mL were defined as the normal reference ranges.

### The influence of gender, age, and sample storage on the detection results of T3-Ab and T4-Ab

**Figure 4:**
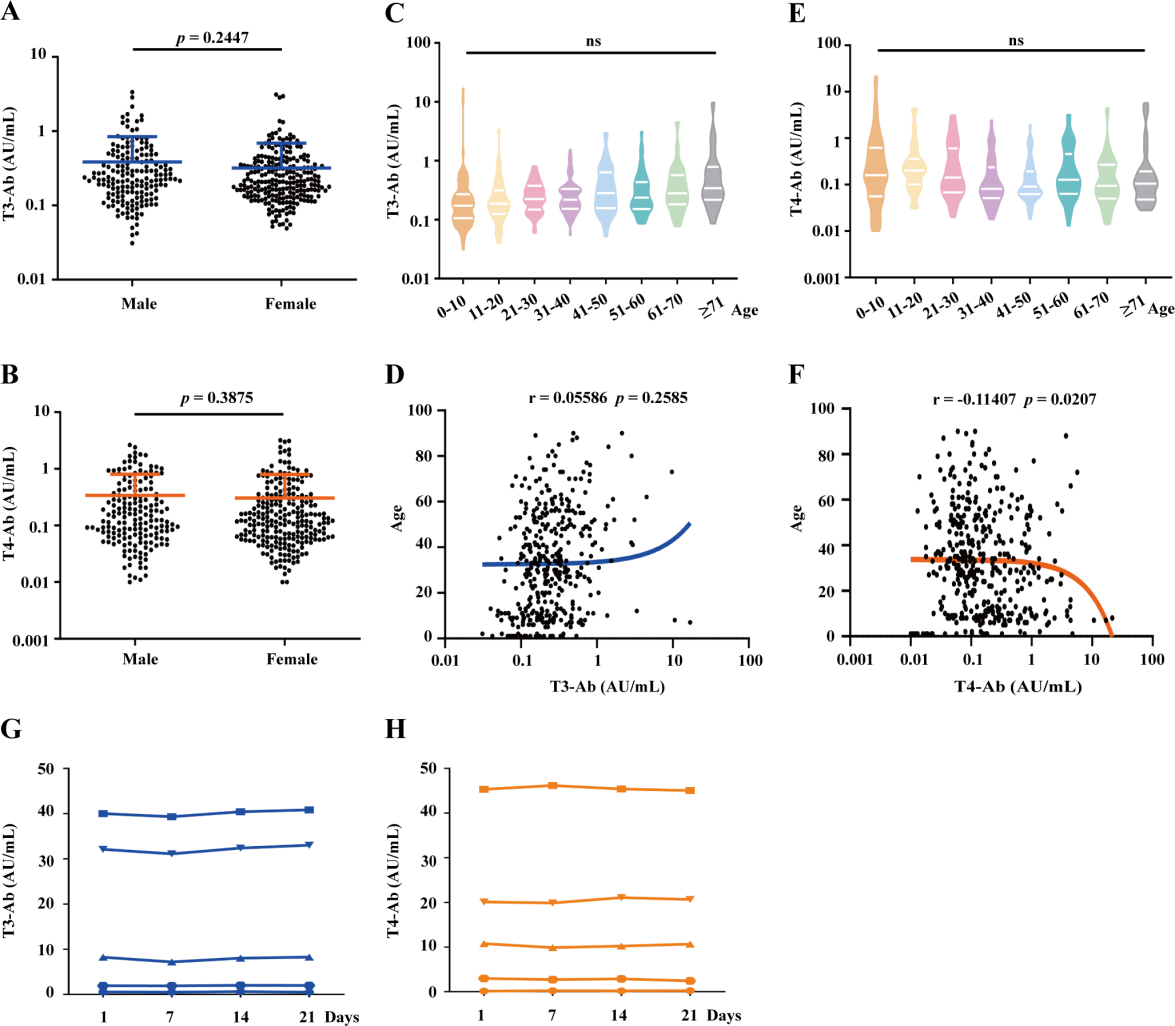
Analysis of T3-Ab and T4-Ab differences based on gender, age, and sample storage conditions. **(A, B)** Scatter plots depict T3-Ab and T4-Ab levels between males and females. **(C, E)** Violin plots showing the distributions of T3-Ab and T4-Ab across different age groups. **(D, F)** Scatter plots illustrating the relationships between T3-Ab or T4-Ab levels and age. **(G, H)** Changes in T3-Ab and T4-Ab levels at 0, 7, 14, and 21 days of storage at 4°C are presented. Statistical analyses utilized the Mann–Whitney U test, ANOVA, and Pearson correlation analysis, with *p* < 0.05 indicating statistical significance.

Our study evaluated the differences in T3-Ab and T4-Ab levels between male and female participants among the 415 healthy controls (173 males, 242 females). Statistical analysis revealed that the T3-Ab concentration in males was 0.49 ± 0.46 AU/mL, whereas in females, it was 0.32 ± 0.37 AU/mL, with no significant difference between the two groups (**Figure 4A**, *p* = 0.2447). For T4-Ab levels, males had a concentration of 0.34 ± 0.47 AU/mL, compared to a concentration of 0.31 ± 0.49 AU/mL in females, values which were not statistically different from one another (**Figure 4B**, *p* = 0.3875). The healthy control group with sample sizes in gender subgroups sufficient to detect medium effect sizes (Cohen’s d = 0.5, power > 95%). However, no significant differences in T3-Ab/T4-Ab concentrations were observed between groups.

Further analysis categorized healthy individuals by age into several groups: 0–10 years, 11–20 years, 21–30 years, 31–40 years, 41–50 years, 51–60 years, 61–70 years, and above 71 years. One-way ANOVA revealed that there were no significant differences in T3-Ab levels across the different age groups (**Figures 4C, F** = 1.208, *p* = 0.2931). A similar pattern was observed for T4-Ab levels (**Figures 4E, F** = 1.810, *p* = 0.0838). Age-stratified analysis covered eight age groups (5-60 cases/group), and although some subgroups had relatively small sample sizes, overall ANOVA revealed no age-related differences (*p* = 0.2931 for T3-Ab, *p* = 0.3421 for T4-Ab). Similarly, correlation analyses of T3-Ab and T4-Ab levels with age revealed no significant associations (**Figure 4D**, r= 0.05586, *p* = 0.2585; **Figure 4F**, r = -0.011407, *p* = 0.0207), suggesting that age had no significant impact on antibody concentrations. To evaluate the repeatability of measuring T3-Ab and T4-Ab concentrations under standard storage conditions, we assessed four samples at different time intervals (0, 7, 14, and 21 days) at 4°C. The results indicated that both T3-Ab and T4-Ab concentrations remained stable for up to 21 days at 4°C, providing guidance for future clinical laboratory testing.

### T3-Ab and T4-Ab independence from thyroid hormones and other autoantibodies

**Figure 5:**
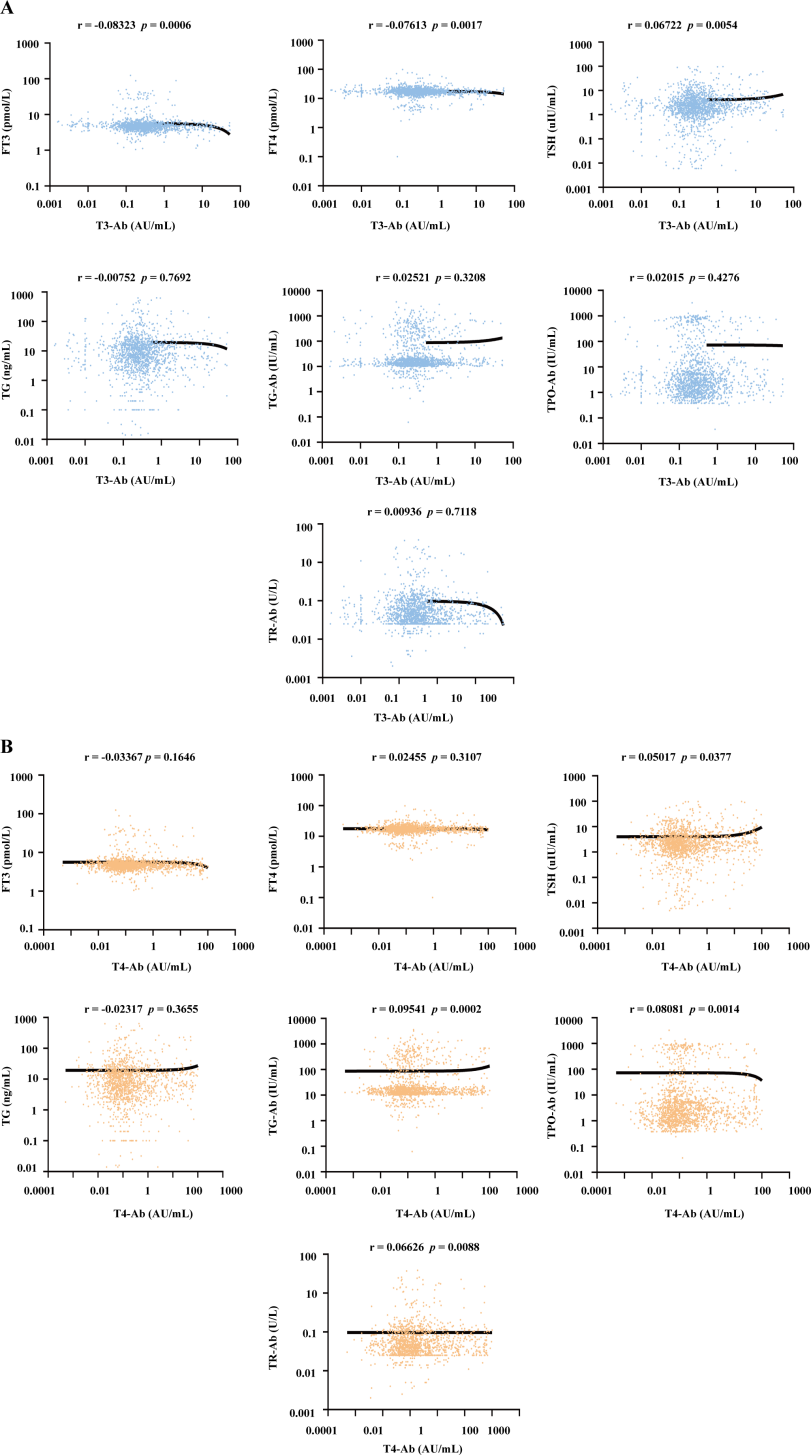
Correlation analysis between serum T3-Ab and T4-Ab levels with thyroid hormones and autoantibodies across all patients. **(A, B)** Scatter plots illustrating the relationships between T3-Ab or T4-Ab levels and FT3, FT4, TSH, TG, TG-Ab, TPO-Ab, or TR-Ab levels (n = 1654). Statistical analysis employs Spearman correlation analysis, with *p* < 0.05 indicating statistical significance.

To investigate the potential associations between T3-Ab/T4-Ab levels and thyroid hormones as well as other related autoantibodies, the serum levels of T3-Ab, T4-Ab, FT3, FT4, TSH, thyroglobulin (TG), thyroglobulin antibody (TG-Ab), thyroid peroxidase antibody (TPO-Ab) and thyroid stimulating hormone receptor antibody (TR-Ab) in 1654 patients were measured via MCLIA, and a correlation analysis of the test results was performed. Serum T3-Ab levels showed no statistically significant correlations with TG (r = -0.00752, *p* = 0.7692), TG-Ab (r = 0.02521, *p* = 0.3208), TPO-Ab (r = 0.02015, *p* = 0.4276), or TR-Ab (r = 0.00936, *p* = 0.7118). By contrast, weak but statistically significant correlations were observed with FT3 (r = -0.08323, *p* = 0.0006), FT4 (r = -0.07613, *p* = 0.0017), and TSH (r = 0.06722, *p* = 0.0054) (**Figure 5A**). Notably, all correlation coefficients exhibited small effect sizes (|r| < 0.1), which, according to Cohen’s guidelines for correlation strength (13), fall within the range of “no practical association”, indicating these correlations are clinically negligible. Furthermore, no practical association was detected between serum T4-Ab levels and FT3, FT4, TSH, or TG levels (**Figure 5B**, all |r| < 0.1, *p* > 0.05). For TG-Ab, TPO-Ab, and TR-Ab, although the *p* values were < 0.05, all |r| remained < 0.1. Our results indicated that T3-Ab and T4-Ab levels exhibit no clinically meaningful correlation with existing thyroid hormones (FT3, FT4, TSH, TG) or autoantibodies (TG-Ab, TPO-Ab, TR-Ab). This suggests that T3-Ab and T4-Ab can serve as independent biomarkers for evaluating thyroid dysfunction, as their utility is not confounded by traditional thyroid markers.

### Impact of T3-Ab and T4-Ab on the FT3 and FT4 test results

**Figure 6:**
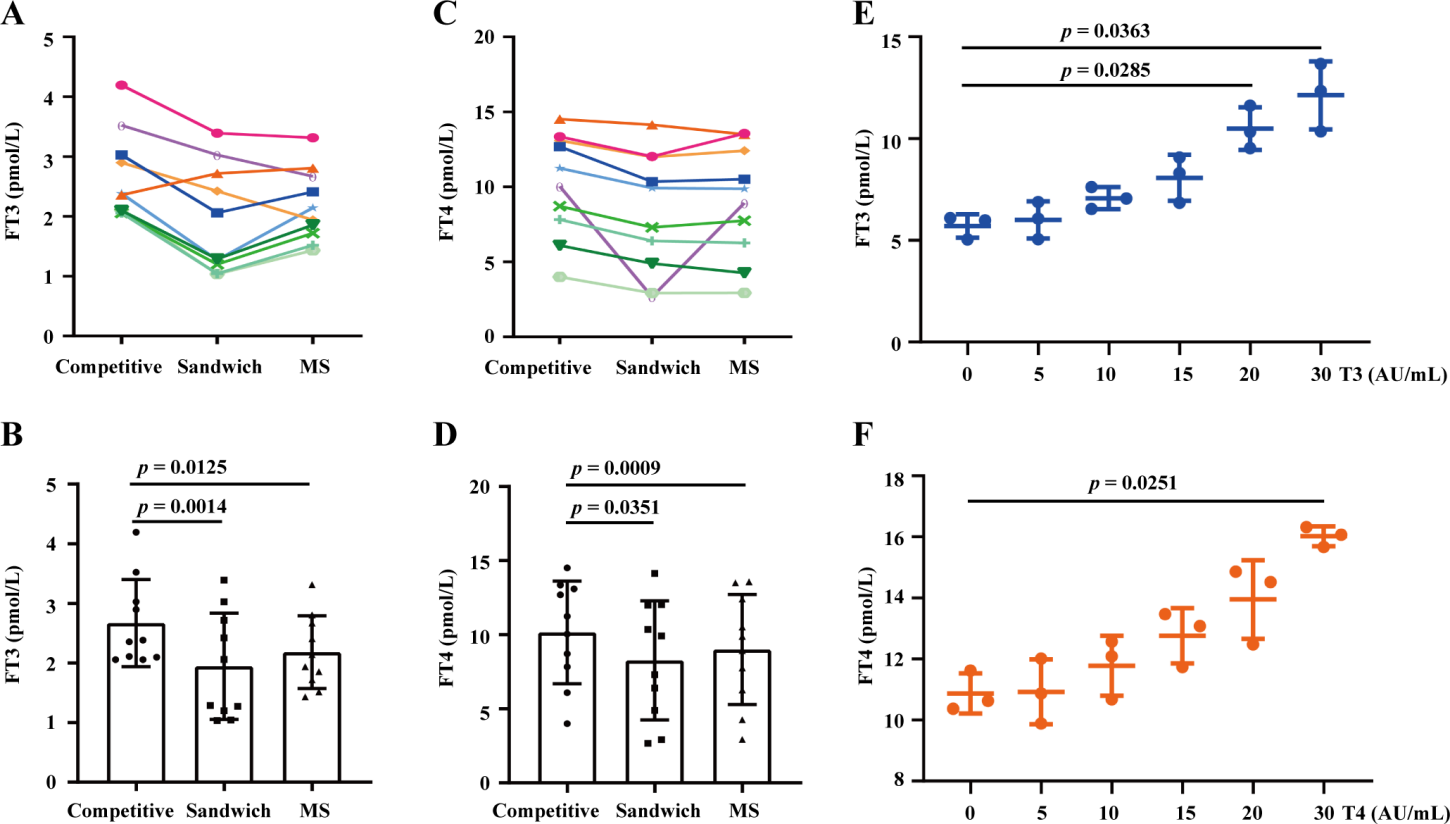
Assessing the impact of T3-Ab and T4-Ab on the FT3 and FT4 test results. **(A, C)** Longitudinal graphs depict FT3 and FT4 levels detected by competitive immunoassay, sandwich immunoassay, and mass spectrometry from serum samples of 10 patients positive for T3-Ab or T4-Ab. **(B, D)** Histograms illustrating the variance observed. **(E, F)** Comparative analysis of FT3 and FT4 measurements with post-supplementation of T3-Ab or T4-Ab standards at different concentrations. Statistical analysis was performed via ANOVA, with significance set at *p* < 0.05.

Current FT3 and FT4 hormone testing methods are based on competitive chemiluminescence immunoassays. To determine whether the presence of T3-Ab and T4-Ab antibodies affects thyroid hormone detection and correlates with detection methods, we randomly selected 10 serum samples from patients positive for T3-Ab or T4-Ab. We utilized competitive chemiluminescence immunoassays, sandwich immunoassays, and mass spectrometry to measure the FT3 and FT4 levels of these patients, and the mass spectrometry results were used as the standard reference. The results indicated that, in competitive immunoassays, 9 out of 10 patients presented significantly higher FT3 levels than did those measured with sandwich assays and mass spectrometry (**Figures 6A, B**, *p*= 0.0014, *p* = 0.0125). Similarly, 9 patients had elevated FT4 levels in the competitive assays compared with those in the sandwich assays and mass spectrometry, where only two patients had FT4 levels that were slightly higher than those measured by mass spectrometry, whereas the remainder were lower (**Figures 6C, D**, *p* = 0.0351; *p* = 0.0009).

To further demonstrate the impact of T3-Ab and T4-Ab on FT3 and FT4 testing, we conducted supplementation experiments and revealed that as the concentrations of T3-Ab and T4-Ab increased, the FT3 and FT4 test results increased significantly at supplementation concentrations of 20 AU/mL and 30 AU/mL (**Figure 6E**, *p* = 0.0285; *p* = 0.0363) and at 30 AU/mL (**Figure 6F**, *p* = 0.0251). These findings suggested that T3-Abs and T4-Abs could cause apparent increases in FT3 and FT4 hormone levels, particularly with respect to the competitive immunoassay principle.

### Distribution levels and positive rates of T3-Ab and T4-Ab in diseases

**Figure 7:**
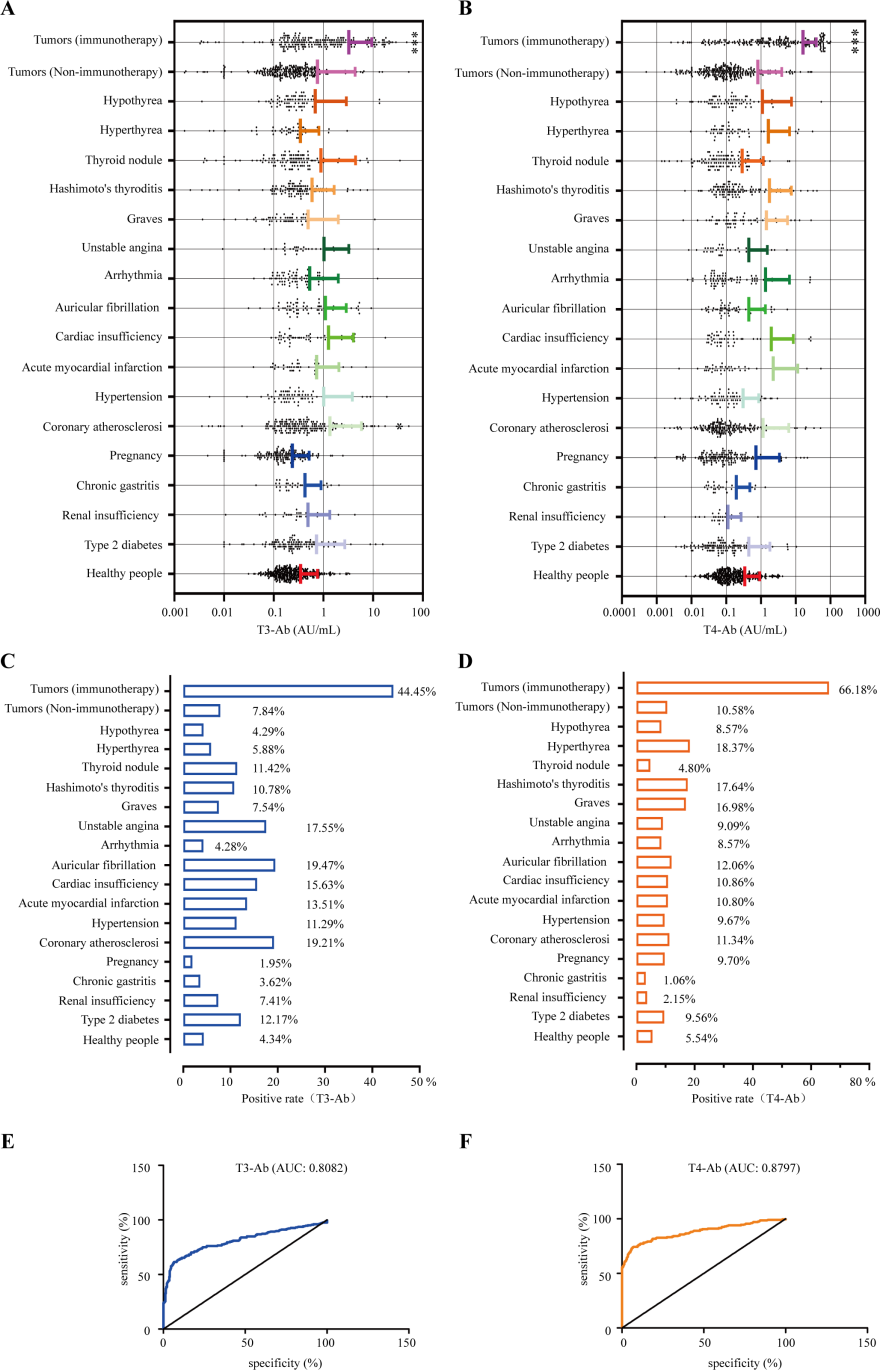
Positive rates of T3-Ab and T4-Ab in the presented diseases. **(A, B)** Distribution levels of T3-Ab and T4-Ab in the indicated disease categories (n = 1654). **(C, D)** Positive rate statistics for T3-Ab and T4-Ab in in the indicated disease categories. **(E, F)** ROC curves assessing the diagnostic performance of T3-Ab and T4-Ab in predicting ICB exposure. Statistical analysis was performed via ANOVA and DeLong’s test, with significance defined as *p* < 0.05.

In this study, we included 1654 patients with the indicated diseases and 415 healthy individuals as a control group, and the serum concentrations of the biomarkers T3-Ab and T4-Ab were measured. The results indicated variable expression levels of T3-Ab and T4-Ab in different diseases. Notably, the T3-Ab level was significantly elevated in patients receiving tumor immune checkpoint blockade (ICB) therapy compared with both the tumor non-immunotherapy group and the healthy control group (ICB therapy group: 3.23 ± 6.13 AU/mL; tumor non-immunotherapy group: 0.75 ± 3.58 AU/mL; healthy control group: 0.34 ± 0.41 AU/mL, *p* < 0.0001. **Figures 7A, B**, **Table 1**). Similarly, T4-Ab levels were significantly higher in the ICB therapy group than that in the tumor non-immunotherapy group and healthy control group (ICB therapy group: 16.16 ± 23.11 AU/mL; tumor non-immunotherapy group: 0.81 ± 3.15 AU/mL; healthy control group: 0.34 ± 0.57 AU/mL, *p* < 0.0001, **Figures 7A, B**, **Table 1**). Additionally, patients with coronary atherosclerosis presented significantly elevated T3-Ab levels compared with healthy controls (coronary atherosclerosis: 1.35 ± 4.47 AU/mL, *p* = 0.0259). Further analysis demonstrated that when both T3-Ab and T4-Ab were established with a threshold of 1.0 AU/mL, the positive rates in the ICB therapy group were 44.44% for T3-Ab and 66.18% for T4-Ab. In contrast, the rates in the tumor non-immunotherapy group were only 7.84% and 10.58%, whereas in the healthy control group, the rates were merely 4.34% and 5.54% (**Figures 7C, D**, **Table 1**).

**Table 1:** The levels and positivity rates of T3-Ab and T4-Ab across different disease groups.

Groups	T3-Ab		T4-Ab	
	Levels (Mean±SD; AU/mL)	Positivity rates (%)	Levels (Mean±SD; AU/mL)	Positivity rates (%)
Tumor (immunotherapy)	3.23 ± 6.13	44.45	16.16 ± 23.11	66.18
Tumor (non-immunotherapy)	0.75 ± 3.58	7.84	0.81 ± 3.15	10.58
Hypothyrea	0.69 ± 2.21	4.29	1.09 ± 6.48	8.57
Hyperthyrea	0.34 ± 0.47	5.88	1.63 ± 5.02	18.37
Thyroid nodules	0.89 ± 3.55	11.42	0.29 ± 0.88	4.80
Hashimoto's thyroiditis	0.59 ± 1.05	10.78	1.76 ± 5.72	17.64
Graves disease	0.49 ± 1.49	7.54	1.44 ± 4.37	16.98
Unstable angina	1.03 ± 2.22	17.55	0.45 ± 1.08	9.09
Arrhythmia	0.53 ± 1.46	4.28	1.35 ± 5.26	8.57
Atrial fibrillation	1.01 ± 1.78	19.47	0.49 ± 0.89	12.06
Cardiac insufficiency	1.26 ± 2.75	15.63	1.99 ± 6.51	10.86
Acute myocardial infarction	0.73 ± 1.32	13.51	2.24 ± 9.25	10.80
Hypertension	1.01 ± 2.82	11.29	0.31 ± 0.55	9.67
Coronary atherosclerosis	1.35 ± 4.47	19.21	1.15 ± 5.17	11.34
Pregnancy	0.24 ± 0.28	1.95	0.72 ± 2.68	9.70
Chronic gastritis	0.47 ± 0.84	3.62	0.19 ± 0.28	1.06
Renal insufficiency	0.49 ± 0.85	7.41	0.11 ± 0.16	2.15
Type 2 diabetes	0.72 ± 1.95	12.17	0.45 ± 1.36	9.56
Healthy people	0.34 ± 0.41	4.34	0.34 ± 0.57	5.54

To evaluate the diagnostic performance of T3-Ab and T4-Ab, receiver operating characteristic (ROC) curve analysis was performed using healthy controls (n = 415) as the negative reference group and ICB-treated tumor patients (n = 207) as the target group. ROC curves demonstrated strong discriminative ability of both antibodies for identifying ICB exposure (**Figures 7E, F**). T3-Ab yielded an AUC of 0.8082 (*p* < 0.0001; 95% CI: 0.78–0.86), with 78.7% sensitivity and 79.3% specificity at the optimal cutoff of 1.2 AU/mL. T4-Ab showed a higher AUC of 0.8797 (*p* < 0.0001; 95% CI: 0.86–0.92), achieving 85.5% sensitivity and 81.2% specificity at a cutoff of 1.9 AU/mL (**Table 2**). These results indicated that T3-Ab and T4-Ab levels were significantly elevated in ICB-treated patients compared to controls, suggesting their potential as biomarkers of ICB exposure or immune activation.

**Table 2:** ROC analysis of T3-Ab and T4-Ab in the diagnosis of ICB induced thyroiditis.

Variables	T3-Ab	T4-Ab
Cut-off (AU/mL)	1.2	1.9
AUC (95%CI)	0.8082 (0.78–0.86)	0.8797 (0.86–0.92)
Sensitivity (%)	78.7	85.5
Specificity (%)	79.3	81.2
*p*	< 0.0001	< 0.0001

## Discussion

The determination of FT3, FT4 and TSH represents widely used diagnostic methods for evaluating thyroid function. However, several factors present in serum samples may non-specifically bind to the detection reagents, thereby interfering with hormone measurements (14–16). The existence of thyroid autoantibodies was first reported in 1956 by Robbins in a patient with papillary thyroid carcinoma who received ^131^I therapy (17). The reported positive rates of T3-Ab and T4-Ab vary significantly between studies, ranging from 0% to 25% (18–20). This discrepancy in prevalence rates may result from differences in study populations and detection methodologies. Previous investigations have primarily employed radioimmunoprecipitation for T3-Ab and T4-Ab detection (21, 22), which has not resulted in standardized detection kits. Therefore, developing novel detection systems for T3-Ab and T4-Ab could be valuable. In our study, we successfully established a detection system for measuring T3-Ab and T4-Ab levels in human serum via an MCLIA analysis system. By identifying these antibodies as relevant indicators, our research paves the way for further exploration into their roles in various thyroid conditions and their potential impact on treatment strategies.

There are various opinions regarding the impact of factors such as gender, age, and sample storage conditions on the detection results of thyroid hormones and autoantibodies (23, 24). Previous studies have indicated that the prevalence of thyroid disease is significantly greater in females than in males, and tends to increase with age (25). Our findings revealed no significant differences or correlations in T3-Ab or T4-Ab levels based on gender or age. Moreover, these antibodies demonstrated stability under routine laboratory sample storage conditions for up to 21 days, providing foundational information for future clinical application and reference value in establishing the use of T3-Abs and T4-Abs as biomarkers. Additionally, T3-Ab and T4-Ab exhibited no significant correlation with other thyroid hormones or autoantibodies, indicating that they can function as independent biomarkers for assessing thyroid diseases.

At present, the detection of FT3 and FT4 relies on a competitive chemiluminescent immunoassay. Our findings indicate that elevated levels of T3-Ab and T4-Ab in serum may interfere with the detection of FT3 and FT4, leading to falsely high results. Although T3-Ab and T4-Ab are less common than other autoantibodies (26), they are the only reported autoantibodies that interfere with thyroid function tests and bind to tracers in various immunoassay systems, resulting in spurious thyroid hormone concentrations that do not accurately reflect patients’ actual thyroid function status (27–29). Therefore, T3-Ab and T4-Ab have broad potential as supplementary monitoring indicators for thyroid function.

By investigating the distribution levels and positive rates of T3-Ab and T4-Ab in different diseases, we unexpectedly discovered their specific clinical application value. Currently, thyroid dysfunction is the most prevalent endocrine-related adverse event associated with ICB therapy, especially with inhibitors targeting programmed death-1 (PD-1) or programmed cell death 1 Ligand 1 (PD-L1), with incidence rates ranging from 7% to 21% (30–32). These thyroid-related adverse reactions typically present as new-onset hypothyroidism or transient thyrotoxicosis, often progressing to persistent hypothyroidism (33). Moreover, there is currently no unified understanding of the pathological mechanisms underlying ICB therapy-induced thyroiditis. Some reports suggest a potential correlation between TPO-Ab and ICB therapy-induced thyroiditis, yet findings remain inconsistent (34, 35).

The core mechanism of thyroid hormone autoantibody production induced by ICB can be summarized as the following pathological cascade, ICBs activate memory T lymphocytes and promote their aberrant proliferation, triggering a sequential immune response (36). Activated T cells release cytotoxic mediators (such as perforin and granzymes) and proinflammatory cytokines (such as IFN-γ and TNF-α), which directly attack thyroid follicular epithelial cells (37, 38). The sustained inflammatory microenvironment not only exacerbates tissue damage but also disrupts cellular integrity, leading to massive ectopic release of intracellularly stored hormone precursors such as thyroglobulin TG, T3 and T4. These exposed autoantigens are processed by antigen-presenting cells, activating B lymphocyte-mediated humoral immune responses, ultimately inducing the production of T3-Ab and T4-Ab. Identifying more suitable biological indicators to evaluate the thyroid adverse reactions induced by ICB therapy is a critical area of investigation. Our results indicated that the positive rates of T3-Ab (44.45%) and T4-Ab (66.18%) in patients with tumors whounderwent ICB therapy was significantly greater than those in the control and other disease groups. Our study is the first to evaluate the diagnostic performance of T3-Ab and T4-Ab in cancer patients receiving ICB therapy using ROC analysis. T3-Ab and T4-Ab levels were significantly elevated in cancer patients receiving ICB therapy compared to healthy controls and non-ICB tumor patients, suggesting their potential as biomarkers of ICB exposure or immune activation in this population. However, longitudinal studies are needed to validate their role in predicting or monitoring ICB-related thyroid dysfunction.

Unlike FT3 and FT4, which are susceptible to pharmacological thyroid hormone influences, T3-Ab and T4-Ab were significantly elevated in cancer patients receiving ICB therapy. This observation suggests their potential utility as predictive biomarkers for ICB-induced thyroid adverse events, thereby aiding clinicians in making more accurate clinical decisions. Notably, immunotherapy agents like nivolumab may cause assay interference in thyroid function tests, potentially leading to misinterpretation of thyroid status (39). In clinical practice, physicians should perform a combined analysis of serum thyroid autoantibodies (including both T3-Ab and T4-Ab) for accurate interpretation of thyroid function test results.

Beyond oncology, our study underscores the multifaceted clinical utility of T3-Ab and T4-Ab. In patients receiving ICB therapy, their positivity rates were significantly higher than those in control groups, indicating that these antibodies may be associated with ICB exposure or immune activation. Combining the monitoring of these antibodies with conventional laboratory tests aids in risk stratification and guides proactive management strategies. In non-cancer populations, these autoantibodies resolve diagnostic challenges by identifying assay interference in individuals with discordant thyroid hormone profiles, thereby preventing misdiagnosis. Notably, their lack of clinical correlation with traditional thyroid markers solidifies their role as independent biomarkers, with potential applications in subtyping autoimmune thyroid disorders and predicting disease progression trajectories.

However, this study has several limitations. We did not comprehensively analyze potential confounding factors (such as medications and biotin supplementation) that may influence test results, requiring further validation in future studies. In addition, although retrospective analysis showed sufficient power to detect medium effect sizes in gender subgroups, some age-stratified groups (such as the 0–10 years group) had limited sample sizes, potentially compromising the ability to identify subtle age-related differences. Prospective studies with larger sample sizes in specific age ranges are needed to clarify age-related effects on T3-Ab/T4-Ab concentrations. Morever, the immunological mechanisms underlying high T3-Ab/T4-Ab secretion in ICB-treated patients remain unclear and require mechanistic exploration. Further data collection and analysis on ICB-related thyroid dysfunction are needed. Finally, the single-center design and lack of longitudinal data limit the generalizability of our findings. Multicenter, randomized controlled trials with longer follow-up periods are essential to accumulate robust datasets and enhance the clinical utility of these biomarkers.

In summary, through this study, we successfully developed a detection kit for human T3-Ab and T4-Ab for the first time and initially confirmed their potential importance in treating thyroid diseases, providing necessary tools for advancing research and clinical care related to thyroid-related disorders.

## Conclusion

We successfully established a novel detection method based on the MCLIA analysis system for the first time, and investigated the significance of T3-Ab and T4-Ab detection in the evaluation of thyroid function. Our findings revealed substantial discrepancies in the positive rates of T3-Ab and T4-Ab across different studies. Research has demonstrated that T3-Ab and T4-Ab can serve as independent biomarkers for assessing thyroid-related diseases. Notably, Notably, the rate of detection for these antibodies significantly increases in patients with tumors who are receiving ICB therapy, suggesting their potential as indicators for monitoring thyroid adverse reactions induced by ICB therapy. This study provides foundational information for future assessments and interventions related to thyroid complications associated with immune checkpoint blockade therapy.

## Data Availability

The raw data supporting the conclusions of this article will be made available by the authors, without undue reservation.
